# Tracing sexual contacts of HIV-infected individuals in a rural prefecture, Eastern China

**DOI:** 10.1186/1471-2458-12-533

**Published:** 2012-07-20

**Authors:** Haijiang Lin, Na He, Yingying Ding, Danhong Qiu, Weiming Zhu, Xing Liu, Tiejun Zhang, Roger Detels

**Affiliations:** 1 Department of Epidemiology, School of Public Health, Fudan University, Shanghai, China; 2 The Key Laboratory of Public Health Safety of Ministry of Education, Shanghai, China; 3 Taizhou City Center for Disease Control and Prevention, Taizhou city of Zhejiang Province, Taizhou, China; 4 Department of Epidemiology, School of Public Health, University of California, Los Angeles, CA, USA

**Keywords:** Contact tracing, Sexual behavior, Sexual networks, HIV testing, HIV infection

## Abstract

**Background:**

Contact tracing is especially useful for identifying an infection with few cases in the population, such as HIV in China. Little such research is available in China.

**Methods:**

Every newly diagnosed HIV case from 2008–2010 in Taizhou Prefecture, Zhejiang Province in China, was invited to participate as an “index case” in a contact tracing survey by providing contact information for up to eight sexual contacts who themselves were approached for voluntary HIV counseling and testing (VCT). Those who tested HIV-positive were then subjected to another contact tracing survey. This process was repeated until no more sexual contacts were reported or tested positive.

**Results:**

A total of 463 HIV-infected individuals were newly identified during the study period, including 338 cases who were identified from routine surveillance programs and 125 cases who were identified from the present contact tracing survey. Among these 463 cases, 398 (86.0%) served as ‘index cases’ in the survey, including 290 (85.8%) out of the 338 cases identified from routine surveillance programs and 108 (86.4%) out of the 125 cases identified from the present survey. These 398 ‘index cases’ reported a total of 1,403 contactable sexual contacts, of whom 320 (22.8%) received HIV testing and 125 (39.1%) tested positive for HIV. Willingness to receive HIV testing was high among spouses and long term heterosexual or homosexual partners but extremely low among casual and commercial sex partners of ‘index cases’. Consistent condom use was rare for all participants. A total of 290 independent sexual network components were constructed, with high complexity.

**Conclusion:**

Contact tracing is useful for identifying new HIV infections from spouses or long term sexual partners of HIV-infected individuals. The complicated sexual networks existing between and beyond HIV-infected persons provide opportunities for rapid spread of HIV in such areas.

## Background

People who are unaware of their infection are at higher risk for transmitting human immunodeficiency virus (HIV) to others and are unable to benefit from HIV treatment and prevention services [1,2]. More than 400,000 persons living with HIV in China are estimated to be unaware of their infections and a substantial proportion of HIV-infected individuals are diagnosed at a late acquired immunodeficiency syndrome (AIDS) stage[3-5], in spite of tremendous efforts in scaling up HIV testing[6,7]. Identifying persons with undiagnosed HIV infection and linking them to medical care and prevention services continues to be a priority for HIV prevention and control in China. To achieve this goal, alternative or supplemental HIV testing strategies for certain high risk yet hard-to-approach populations are emergently needed.

Contact tracing, a known strategy for controlling the spread of sexually transmitted infections (STIs), is especially useful for identifying an STI with few cases in the population such as HIV in China[8]. With the assistance of an HIV-positive individual contact tracing allows mapping as many “sexual contacts” as possible, and encouraging them to participate in HIV testing and treatment[1,9-11]. Each infected contact can then become the starting point for new contact tracing, until no more contacts can be found. Such a behavioral network strategy has been used successfully in some countries to identify undiagnosed cases of syphilis and HIV[1,2,9,10,12]. However, few studies using contact tracing strategy for identifying new HIV infections in China have been reported in the English literature.

Traditional epidemiological studies of HIV/STIs have typically focused on individual risk factors [13,14]. As HIV/STI depends on intimate contact to propagate, it is reasonable to believe that inclusion of characteristics about an individual’s behavioral network may be valuable for identifying transmission networks within the population [14-16]. Contact tracing for several cycles beyond the original infected individual facilitates identification and access to behavioral networks of additional individuals at high risk for HIV [10,17,18] and documenting these networks improves understanding of HIV transmission dynamics among study participants and increases the effectiveness of HIV/AIDS control efforts [16,19].

The present study, by tracing sexual contacts of HIV-infected persons, aims to identify new HIV infections that might otherwise go unrecognized by routine practices, evaluate the effectiveness and feasibility of such investigation as a supplemental strategy for HIV identification, and to elucidate sexual network characteristics among HIV-infected individuals and their sexual contacts in Taizhou Prefecture, Eastern China.

## Methods

### Study site and determination of HIV transmission route

This study was conducted in Taizhou Prefecture of Zhejiang Province, Eastern China, with a population of 5.8 million, where a total of 656 HIV/AIDS cases had been reported by the end of 2010. Among them, 67.0% were infected heterosexually, 12.0% infected homosexually and 9.7% infected through injection drug use. The majority (76.0%) of them were identified within the past three years. HIV transmission route was determined upon the judgment of attending health professionals according to the following criteria. First, a HIV-infected individual was considered to be sexually transmitted only if he/she reported having never had any experience of injection drug use and blood/plasma donation or transfusion. Second, for a HIV-infected female, she was considered to be heterosexually transmitted if she reported having had unprotected sex with a HIV-positive spouse or having engaged in commercial or non-commercial extramarital sex. Third, for a HIV-infected male, he was considered to be homosexually transmitted if he reported having had unprotected oral and/or anal sex with another man. Otherwise, he was considered to be heterosexually transmitted.

### Study participants and data collection

**Figure 1:**
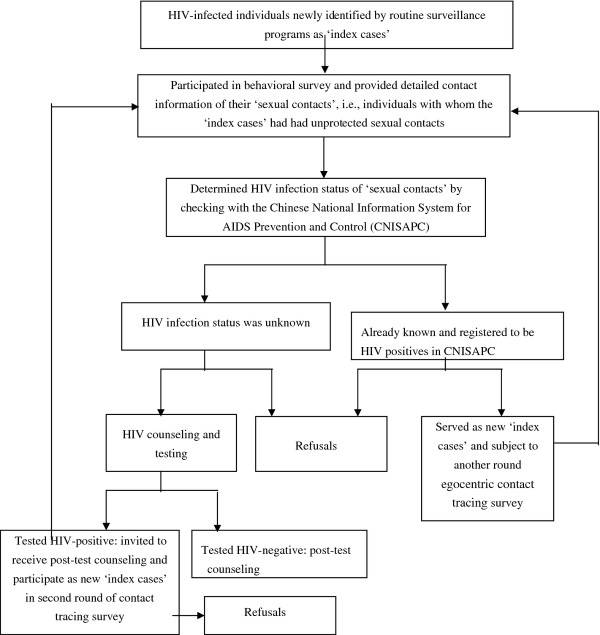
Schematic diagram of the study design and contact tracing procedures.

Each newly reported HIV-infected individual from 2008 through 2010 in Taizhou was invited and assured of the confidentiality of their information in the informed consent to participate as an index case in an egocentric contact tracing survey, which requested numbers, types and contact information about sexual contacts with whom the HIV-infected index case had had sex in the past 12 months. Each participant was encouraged to elicit information on a maximum of eight sexual contacts who were approached and consented to participate in this egocentric contact tracing survey and to receive voluntary HIV counseling and testing (VCT). Information on type of the sexual contact, contact information, HIV status of the sexual contact and condom use for sex with the sexual contact in the past 12 month was collected in a paper-pencil questionnaire interview administered by trained health professionals. The questionnaire was anonymous with a unique identification number linkable to the HIV-infected participant. A sexual contact who tested positive for HIV was then invited to participate as an ‘index case’ in a new contact tracing survey [1,8,20]. This process continued until no more sexual contacts tested HIV positive or no more sexual contacts were reported (Figure 1).

To promote provision of contact information of sexual contacts, HIV-infected index cases were attended and counseled by the researchers, with particular emphases on the following: (1) the importance of early identification of HIV infection and protection of sexual contacts from HIV transmission, (2) national policies about the availability of free HIV counseling, testing, follow-up of disease status, antiretroviral treatment (ART), and if appropriate, prevention for mother-to-child transmission, (3) law enforcement of confidentiality of their HIV infection status, (4) assurance of confidentiality and privacy of the contact information of sexual contacts, and (5) nondisclosure of their names to their reported sexual contacts including those who participated in the next round contact tracing survey.

All identified HIV-infected participants were registered into the Chinese National Information System for AIDS Prevention and Control (CNISAPC), received regular follow-up and health care according to the national guidelines, and if indicated, received free antiretroviral treatment (ART). Each participant received thirty Chinese Yuan (about US$5) for travel reimbursement. The study was approved by the Institutional Review Board (IRB) of Fudan University, Shanghai, China.

### HIV counseling and testing

Sexual contacts of HIV-infected persons who were willing to participate in the study received a face-to-face pre-test counseling administered by a health professional. Usually the pre-test counseling lasted for about 30 to 45 minutes. Venous blood was then collected by an experienced nurse using sterilized needles and tubes. Plasma samples were coded by unique identification numbers and were screened for HIV antibody using an enzyme-linked immunosorbent assay (ELISA; Vironostika HIV Uni-Form II plus O ELISA Kit, Biomerieux, Boxtel, Netherland), according to the manufacturer’s instructions. Those screened to be HIV positive were further confirmed by western blotting (Genelabs Diagnostic, Singapore). All participants, no matter what the HIV testing results were, received routine post-test counseling and referral for other appropriate health services.

### Statistical and network analysis

In addition to descriptive analyses, tests of associations between two categorical variables were based on the Chi-squared test or Fisher’s exact test, as appropriate. A significance level of 0.05 was used for all tests. Sexual network metrics and diagrams were generated using the software programs UCINET 6.0 (Borgatti, S.P., Everett, M.G. and Freeman, L.C. 2002. Harvard, MA: Analytic Technologies) and Pajek 1.09 (Vladimir Batagelj and Andrej Mrvar, University of Ljubljana). Contact tracing proficiency was assessed by calculating a sexual network index (i.e., number of sexual contacts recruited divided by number of recruiters, i.e., HIV-infected ‘index cases’) and by calculating prevalence of newly identified HIV infections among various types of sexual contacts [1,2]. The average degree of the network was assessed by all degrees of every HIV infection summed and divided by the total number of HIV infections in the network.

## Results

### Contact tracing

**Figure 2:**
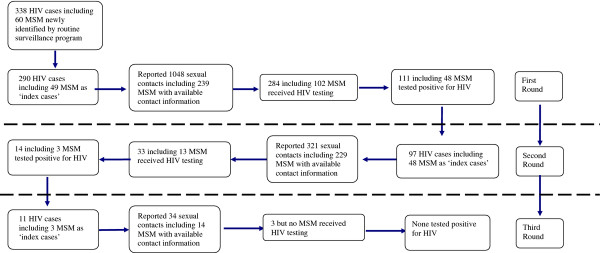
Flow chart of tracing contacts of HIV cases in Taizhou, Eastern China, 2008–2010.

The whole process of contact tracing and identification of HIV infections is summarized in Figure 2 and Table 1. In the first round, 290 (85.8%) out of 338 HIV-infected individuals who were identified from routine HIV surveillance programs served as ‘index cases’ and reported a total of 1,048 sexual contacts with contact information. Of this group of 1,048, 284 (27.1%) received HIV testing, of whom 111 (39.1%) tested positive for HIV. Ninety-seven (87.4%) out of these 111 newly identified HIV infections participated as’index cases’ in the second round of contact tracing survey. They reported a total of 321 sexual contacts with contact information. Of them, only 33 (10.3%) received HIV testing of whom 14 (42.4%) tested positive for HIV. Eleven (78.6%) out of the 14 newly identified HIV cases participated as ‘index cases’ in the third round of contact tracing survey. They reported a total of 34 sexual contacts with contact information. Of them only 3 (8.8%) received HIV testing, but none tested positive for HIV (Table 1).

**Table 1:** HIV testing and infection among sexual contacts of index HIV cases in Taizhou prefecture, Eastern China, 2008-2010

**The process of tracing contacts**	**First round**			**Second round**			**Third round**			**Total**		
	**Heterosexually infected**	**Homosexually infected**	**Total**	**Heterosexually infected**	**Homosexually infected**	**Total**	**Heterosexually infected**	**Homosexually infected**	**Total**	**Heterosexually infected**	**Homosexually infected**	**Total**
HIV cases identified by routine surveillance programs or last round contact tracing survey	278 ^a^	60 ^a^	338 ^a^	63 ^b^	48 ^b^	111 ^b^	11^d^	3 ^d^	14 ^d^	352	111	463
Number and proportion (%) of reported HIV cases participating in contact tracing survey as ‘index cases’	241 (86.7)	49 (81.7)	290 (85.8)	49 (77.8)	48 (100.0)	97(87.4)	8 (72.7)	3 (100.0)	11 (78.6)	298 (84.6)	100 (90.1)	398(86.0)
Number of sexual contacts reported by ‘index cases’	809	239	1048	92	229	321	20	14	34	921	482	1403
Number and proportion (%) of sexual contacts receiving HIV testing	182 (22.5)	102 (42.7)	284 (27.1)	20 (21.7)	13 (5.7)	33 (10.3)	3 (15.0)	0 (0.0)	3 (8.8)	205 (22.3)	115 (23.9)	320 (22.8)
Number and proportion (%) of sexual contacts newly tested HIV positive	62^b^ (34.1)	49^b,c^ (48.0)	111^b^ (39.1)	11^d^ (55.0)	3^d^ (23.1)	14^d^ (42.4)	0	0	0	73 (35.6)	52 (45.2)	125 (39.1)

a: HIV cases identified by routine surveillance programs; b: HIV cases identified by the first round contact tracing survey; c: One of the newly tested HIV positives was the spouse of an HIV-infected MSM; d: HIV cases identified by the second round contact tracing survey.

In sum, a total of 463 HIV-infected individuals were newly reported during the study period, including 338 cases who were identified from the routine surveillance programs and 125 cases who were identified from the present three rounds contact tracing survey. Among these 463 cases, 398 (86.0%) served as ‘index cases’ in the survey, including 290 (85.8%) out of the initial 338 cases identified from routine surveillance programs and 108 (86.4%) out of the 125 cases newly identified from the present survey. These 398 ‘index cases’ reported a total of 1,403 sexual contacts with contact information, of whom none was reported by different ‘index cases’ and appeared in multiple rounds in this study and only 320 (22.8%) actually received HIV testing. Among the 320 contacts who actually received HIV testing, 125 (39.1%) tested positive for HIV. The sexual network index (i.e., the number of sexual contacts recruited divided by the number of recruiters or HIV-infected ‘index cases’) is 0.80 (320/398).

Moreover, the 398 HIV-infected participants or ‘index cases’ and the 64 HIV-infected nonparticipants or refusals were not significantly different by gender, age and HIV transmission route, but significantly different by ethnicity, marital status and official residency. Compared with participants, refusals had a higher proportion of ethnic minorities (26.2% vs. 4.8%), divorced/widowed (23.1% vs. 9.0%), and non-local residents (68.7% vs. 29.1%).

### Sociodemographic and sexual behavioral characteristics of HIV-infected index cases

#### Sociodemographic characteristics

Among the total of 398 HIV-infected index cases, 298 (74.9%) were infected through heterosexual contacts and 100 (25.1%) infected through homosexual contacts, 73.9% were males, 20.6% aged less than 25 years, 95.2% were ethnic Han, 29.1% were never married, and 29.1% were non-local residents (Table 2). Heterosexually and homosexually infected index cases were significantly different by gender, age, ethnicity and marital status (Table 2).

**Table 2:** Sociodemographic and sexual behavioral characteristics of index HIV cases in Taizhou prefecture, Eastern China, 2008-2010

**Characteristics**	**Heterosexually infected**		**Homosexually infected**		**Total**	
	**n**_**1**_ **= 298**	**Proportion (%)**	**n**_**2**_ **= 100**	**Proportion (%)**	**n**_**3**_ **= 398**	**Proportion (%)**
Gender (*χ*^2^ = 47.24, *P* < 0.001)						
Male	194	65.1	100	100.0	294	73.9
Female	104	34.9	—	—	104	26.1
Age (years) (*χ*^2^ = 29.97, *P* < 0.001)						
18-25	50	16.8	32	32.0	82	20.6
26-35	94	31.5	46	46.0	140	35.2
36-45	78	26.2	16	16.0	94	23.6
≥46	76	25.5	6	6.0	82	20.6
Ethnicity (*χ*^2^ = 6.695, *P* = 0.010)						
Han	279	93.6	100	100.0	379	95.2
others	19	6.4	0	0.0	19	4.8
Marital status (*χ*^2^ = 37.6, *P* < 0.001)						
Currently Married	200	67.1	42	42.0	242	60.8
Never married	63	21.1	53	53.0	116	29.1
Divorced or widowed	31	10.4	5	5.0	36	9.0
Unknown	4	1.3	0	0.0	4	1.0
Official residency (*χ*^2^ = 1.524, *P* = 0.218)						
Local (i.e., study area)	216	72.5	66	66.0	282	70.9
Non-local	82	27.5	34	34.0	116	29.1
Ever had non-commercial casual heterosexual partners or sexual contacts in the past 12 months (*χ*^2^ = 21.27, *P* < 0.001)						
Yes	80	26.8	5	5.0	85	21.4
No	218	73.2	95	95.0	313	78.6
Ever had commercial heterosexual partners or sexual contacts in the past 12 months (*χ*^2^ = 188.6, *P* < 0.001)						
Yes	233	78.2	0	0.0	233	58.5
No	65	21.8	100	100.0	165	41.5
Ever had long-term homosexual partners or sexual contacts in the past 12 months						
Yes	—	—	91	91.0	91	91.0
No	—	—	9	9.0	9	9.0
Ever had non-commercial casual homosexual partners or sexual contacts in the past 12 months						
Yes	—	—	80	80.0	80	80.0
No	—	—	20	20.0	20	20.0
Ever had commercial homosexual partners or sexual contacts in the past 12 months						
Yes	—	—	63	63.0	63	63.0
No	—	—	37	37.0	37	37.0
Total number of sexual partners or sexual contacts in the past 12 months (*χ*^2^ = 26.04, *P* < 0.001)						
1	52	17.4	0	0.0	52	13.1
2-4	50	16.8	27	27.0	77	19.3
5-9	57	19.1	29	29.0	86	21.6
10-19	85	28.5	23	23.0	108	27.1
20 or more	54	18.1	21	21.0	75	18.8
Condom use for sex with sexual contacts in the past 12 months (*χ*^2^ = 1.673, *P* = 0.196)						
Consistently	0	0.0	0	0.0	0	0.0
Inconsistently	200	67.1	60	60.0	260	65.3
Never	98	32.9	40	40.0	138	34.7
Reported having sexual partners or sexual contacts with known HIV positive status (*χ*^2^ = 0.312, *P* = 0.576)						
Yes	19	6.4	8	8.0	27	6.8
No	279	93.6	92	92.0	371	93.2

n_1_ number of index HIV cases infected heterosexually; n_2_: number of index HIV cases infected homosexually; n_3_: total number of index cases.

#### Sexual behaviors in the past 12 months

About 26.8% (80/298) of heterosexually infected index cases and 5.0% (5/100) of homosexually infected index cases had ever had non-commercial casual heterosexual partners in the past 12 months, whereas the majority (78.2%) of heterosexually infected index cases but none homosexually infected index cases had had commercial heterosexual partners (Table 2). Most (91.0%) of homosexually infected index cases had long-term homosexual partners, and a substantial proportion of them had non-commercial casual homosexual partners (80.0%) or commercial homosexual partners (63.0%). Homosexually infected index cases reported more contactable sex partners than those heterosexually infected (Table 2).

None of the index cases reported consistent condom use for sex with their reported sexual contacts in the past 12 months, and 34.7% had never used condoms for sex with their reported sexual contacts (Table 2). Condom use was not significantly different by HIV transmission route of index cases (Table 2). Twenty-seven (6.8%) index cases reported having HIV positive partners with known HIV positive status in the past 12 months.

#### Sexual networks in the past 12 months

**Figure 3:**
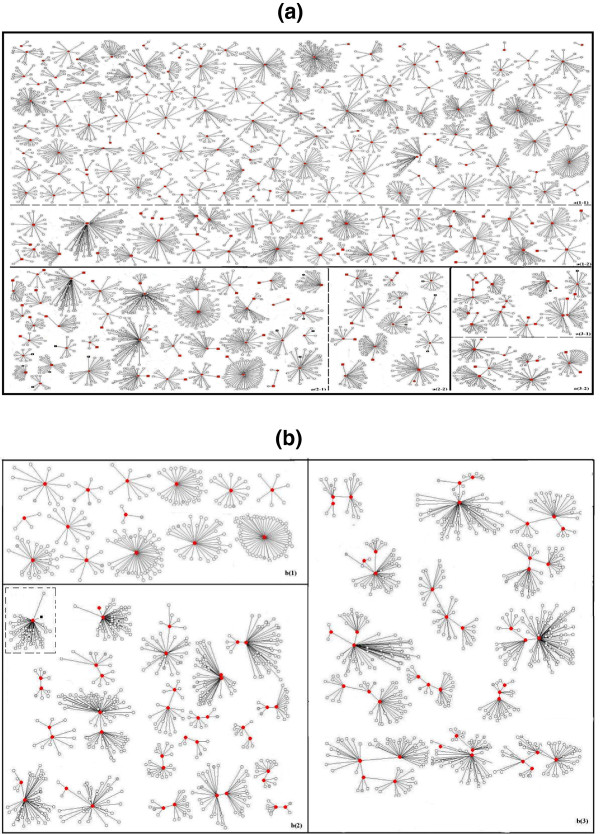
** Sexual networks (n = 290) of HIV-infected participants in Taizhou prefecture, Eastern China, 2008–2010 (with the average degree of network 13.0).(a)** Sexual networks (n = 241) of heterosexually infected HIV positive participants(with the average degree of network 12.2(rang from 1 to 81)): a(1–1) sexual networks (n = 131) of male HIV positive participants with none of risky contacts tested HIV positive; a(1–2) sexual networks (n = 49) of female HIV positive participants with none of risky contacts tested HIV positive; a(2–1) sexual networks (n = 37) of male HIV positive participants with one of risky contacts tested HIV positive; a(2–2) sexual networks (n = 12) of female HIV positive participants with one of risky contacts tested HIV positive; a(3–1) sexual networks (n = 8) of male HIV positive participants with two of risky contacts tested HIV positive; a(3–2) sexual networks (n = 4) of female HIV positive participants with two of risky contacts tested HIV positive. **(b)** Sexual networks (n = 49) of homosexually infected HIV positive participants (i.e., HIV-infected MSM) (with the average degree of network 15.3(rang from 2 to 80)): b(1) sexual networks (n = 14) of HIV-infected MSM with none of risky contacts tested HIV positive; b(2) sexual networks (n = 11) of HIV-infected MSM with one of risky contacts tested HIV positive. The one inside the small dashed square represents the network of an HIV-infected MSM whose wife tested HIV positive. b(3) sexual networks (n = 24) of HIV-infected MSM with two or more of risky contacts tested HIV positive. *Red or dark dots* represent male HIV positive participants; *Red or dark squares* represent female HIV positive participants; *Grey dot*s represent risky contacts who tested HIV negative in the present survey; *Circles* represent risky contacts who did not receive HIV testing. *Hollow dots or squares* represent HIV-infected individuals who refused to participate in the present survey.

A total of 290 independent sexual networks were constructed for index cases and their reported sexual contacts, including 241 (83.1%) for heterosexually infected index cases and 49 (16.9%) for homosexually infected index cases which included twenty-nine (59.2%) sexual networks involving both heterosexual and homosexual partners or contacts (Figure 3). Of the 290 sexual networks, 18 (6.2%) were with two members, 31 (10.7%) with three to five members, 55 (18.9%) with six to ten members, 99 (34.1%) with eleven to twenty members, and 87 (30.0%) with twenty-one to as many as eighty-two members. The average degree (i.e., the number of sexual contacts) of the sexual network was 13.0 (ranging from 1 to 81) overall, 12.2 (ranging from 1 to 81) for heterosexually infected index cases and 15.3 (ranging from 2 to 80) for homosexually infected index cases, respectively.

### HIV testing and infection among sexual contacts by sexual behavioral characteristics in the past 12 months

#### HIV testing and infection by types of sexual relationship

The proportion of sexual contacts who received HIV testing varied by different types of sexual relationship between index cases and their sexual contacts, very high among spouses or long-term heterosexual partners (81.4%, 192/236) of heterosexually infected index cases and long-term homosexual partners (62.9%, 90/143) of homosexually infected index cases, but very low among casual or commercial sex partners (Table 3). Among those receiving HIV testing, the proportion of testing HIV positive was 53.3% (48/90) among long-term homosexual partners of homosexually infected index cases and 38.0% (73/192) among spouses or long-term heterosexual partners of heterosexually infected index cases (Table 3). In addition, 3 (75.0%) out of 4 commercial homosexual partners tested positive for HIV.

**Table 3:** HIV testing and infection among sexual contacts of index HIV cases by sexual behavioral characteristics in Taizhou prefecture, Eastern China, 2008–2010

**Sexual behavioral characteristics**	**Heterosexually infected (N**_**1**_ **= 298)**			**Homosexually infected (N**_**2**_ **= 100)**			**Total (N = 398)**		
	**Sexual contacts with contact information (No., %)**	**Number and proportion of sexual contacts receiving HIV testing (No., %)**	**Number and prevalence of sexual contacts tested HIV + (No., %)**	**Sexual contacts with contact information (No., %)**	**Number and proportion of sexual contacts receiving HIV testing (No., %)**	**Number and prevalence of sexual contacts tested HIV + (No., %)**	**Sexual contacts with contact information (No., %)**	**Number and proportion of sexual contacts receiving HIV testing (No., %)**	**Number and prevalence of sexual contacts tested HIV + (No., %)**
	**921, 100%**	**205, 22.3**	**73, 35.6**	**482, 100%**	**115, 23.9**	**52, 45.2**	**1403, 100%**	**320, 22.8**	**125, 39.1**
**Sexual relationship between index cases and their sexual contacts in the past 12 months**								(*χ*^*2*^ = 809.70, *P*<0.001)^**a**^	**c**
Spouses or long-term heterosexual partners	236, 25.6	192, 81.4	73, 38.0	48, 10.0	20, 41.7	1, 5.0	284, 20.2	212, 74.6	74, 34.9
Non-commercial casual heterosexual partners	111, 12.1	7, 6.3	0	5, 1.0	0	0	116, 8.3	7, 6.0	0
Commercial heterosexual partners	574, 62.3	6, 1.0	0	0	0	0	574, 40.9	6, 1.0	0
Long-term homosexual partners	—	—	—	143, 29.7	90, 62.9	48, 53.3	143, 10.2	90, 62.9	48, 53.3
Non-commercial casual homosexual partners	—	—	—	180, 37.3	1, 0.6	0	180, 12.8	1, 0.6	0
Commercial homosexual partners	—	—	—	106, 22.0	4, 3.8	3, 75.0	106, 7.6	4, 3.8	3, 75.0
**Condom use between index cases and their sexual contacts in the past 12 months**								(*χ*^*2*^ *=* 4.95, *P =* 0.084)^**a**^	(*χ*^*2*^ *=* 15.32, *P<*0.001)^**b**^
Consistently	84, 9.1	15, 17.9	0	32, 6.6	4, 12.5	0	116, 8.3	19, 16.4	0
Inconsistently	269, 29.2	57, 21.2	19, 33.3	176, 36.4	37, 21.0	14, 37.8	445, 31.7	94, 21.1	33, 35.1
Never	568, 61.6	133, 23.4	54, 40.6	274, 56.9	74, 27.0	38, 51.4	842, 60.0	207, 24.6	92, 44.4
**Size (i.e., number of members) of sexual networks constructed for index HIV cases and their sexual contacts in the past 12 months**								(*χ*^*2*^ *=* 27.62, *P*<0.001)^**a**^	(*χ*^*2*^ *=* 16.05, *P =* 0.013)^**b**^
2	18, 2.0	12, 66.7	1, 8.3	0	0	0	18, 1.3	12, 66.7	1, 8.3
3	15, 1.6	6, 40.0	0	3, 0.6	1, 33.3	0	18, 1.3	7, 38.9	0
4	44, 4.8	6, 13.6	1, 16.7	0	0	0	44, 3.1	6, 13.6	1, 16.7
5	16, 1.7	2, 12.5	0	0	0	0	16, 1.1	2, 12.5	0
6-10	193, 21.0	36, 18.7	12, 33.3	53, 11.0	13, 24.5	5, 38.5	246, 17.5	49, 19.9	17, 34.7
11-20	354, 38.4	76, 21.5	28, 36.8	98, 20.3	21, 21.4	9, 42.9	452, 32.2	97, 21.5	37, 38.1
>20	281, 30.5	67, 23.8	31, 50.8	328, 68.0	80, 24.3	38, 47.5	609, 43.4	147, 24.1	69, 48.9

**a**: Chi-square test comparing proportions of sexual contacts receiving HIV testing by sexual behavioral characteristics. **b**: Chi-square test comparing proportions of sexual contacts tested HIV positive by sexual behavioral characteristics. **c**: Chi-square test was not applicable due to a high proportion of cells having expected value < 5.

#### HIV testing and infection by condom use

As shown in Table 3, the proportion of sexual contacts receiving HIV testing did not vary by condom use. However, the proportion of sexual contacts tested to be HIV positive was significantly different by condom use and was higher (44.4%, 92/207) among those having never used condoms than those using condoms inconsistently (35.1%, 33/94). None of sexual contacts with consistent condom use were HIV infected.

#### HIV testing and infection by size of sexual networks

Both proportions of sexual contacts receiving HIV testing and those tested to be HIV positive varied significantly by size of sexual networks constructed for index HIV cases and their sexual contacts (Table 3). The proportion of sexual contacts tested HIV positive was much higher among those involved in larger sexual networks.

## Discussion

To the best of our knowledge, this is the first study using a behavioral network strategy to identify undiagnosed HIV infections in an area with relatively low prevalence of HIV in China. The study identified many HIV-infected individuals including HIV-infected men having sex with men (MSM) who might have otherwise remained undiagnosed, suggesting that tracing sexual contacts of newly reported HIV cases is very helpful in identifying new HIV infections and in better understanding and controlling the HIV epidemic. The high percentages of spouses or long-term heterosexual and homosexual partners receiving HIV testing as well as testing HIV positive indicate that contact tracing, as an HIV testing and identification strategy, is extremely feasible and effective for such high risk groups. Furthermore, a large number of sexual contacts of HIV-infected individuals were identified to be at high risk of HIV infection,but were reluctant to accept HIV testing for the fear of stigma or discrimination or the low awareness of HIV risks[21]. This underscores the importance of better promoting HIV counseling and testing among these people.

Nevertheless, very few casual or commercial sex partners were able to be contacted or were willing to receive HIV testing. With unknown HIV infection status, they were very likely to continue to engage in risky behaviors and therefore to be at continued high risk of HIV infection or transmitting HIV, if infected. This is particularly relevant for commercial homosexual partners or male sex workers as a very high proportion of HIV positives were observed among the few receiving HIV testing. Male sex workers or so-called ‘money boys’ have been identified as high risk group for HIV infection in China [22,23].

The construction of sexual networks for all HIV positive participants and their sexual contacts offers a unique opportunity for visually monitoring potential threats and patterns of HIV spreading in an area and for examining an individual’s role in HIV transmission at a population dynamic level instead of the individual level which typically asks for personal independent risk behaviors or exposures to the disease. Individuals who have same individual risks may play different roles in HIV transmission depending on patterns of their connections with other individuals in the network, and a group of individuals behave as a population system when patterns of connections among these individuals having HIV related behavior influence population health outcomes [14-16,24,25]. In this study, a substantial proportion of sexual networks were mapped out which had two or more HIV-infected participants connected with each other in same networks. This was particularly obvious for sexual networks involving MSM, suggesting a complicated and dynamic chain of HIV sexual transmission especially among homosexuals.

Moreover, the complexity of the sexual networks of HIV-infected individuals implies that much more effort is urgently needed to reach those at high risk of HIV transmission or HIV infection, to intervene and reduce risky behaviors and most importantly to break unprotected or risky sexual networks among them. Compared with simply changing individual risk behaviors, changing the pattern of behavioral connections between HIV positive and HIV negative individuals through behavioral network-based preventive measures may have more impact on HIV epidemic at population level, which provides a new strategy for developing and implementing community-based prevention programs [14,26-28].

Despite the promising findings presented here, several limitations need to be addressed. First, this study was based in a prefecture in Eastern China where the HIV epidemic is relatively low and primarily sexual. It turned out that all HIV-infected participants in the study were sexually infected and none of them reported having used drugs. Nonetheless, the study results might not be generalizable to other sites or circumstances where injection drug use is not an ignorable transmission route of HIV. Therefore, future studies should be conducted in other settings such as large cities and highly epidemic areas in Western and Southern China, and should collect thorough information on both sexual and non-sexual risk behaviors such as drug use so as to elucidate more completely the risk behavioral network characteristics of HIV transmission. Second, to be practicable, sexual contacts and sexual networks were traced and measured only for the past 12 months in this exploratory study. As sexual networks are dynamic and tend to change over time, the cross-sectional nature of this study creates only a snapshot of sexual networks of HIV-infected people in the study area. The inability to ascertain dates of risky behaviors and HIV seroconvertion made it impossible to assess HIV transmission dynamics in this study, and provides a rationale for future longitudinal studies.

## Conclusions

Despite the limitations, this study found that contact tracing is a feasible and effective strategy to identify new HIV infections including high risk spouses and long term sexual partners of HIV-infected individuals. However, it seems extremely difficult to trace commercial sexual partners or casual sexual partners on their HIV infection status. The complicated sexual networks existing between and beyond HIV-infected persons demonstrate the great potentials of HIV spreading in such areas. Future research and programmatic efforts should substantiate and extend these findings and begin to focus on strategies that can address both individual and network or population risk.

## Abbreviations

HIV, Human immunodeficiency virus; AIDS, Acquired immunodeficiency syndrome; VCT, Voluntary HIV counseling and testing; STIs, Sexually transmitted infections; ART, Antiretroviral treatment; ELISA, Enzyme-linked immunosorbent assay; MSM, Men having sex with men.

## Competing interests

The authors declare that they have no competing interests.

## Authors’ contributors

HL contributed to study design, data collection, data analysis and drafting the study report including all tables and figures. NH designed and generally supervised the study, guided data analysis and data interpretation, drafted and edited the manuscript. YD, DQ, WZ, XL and TZ contributed to participant recruitment, data collection and data interpretation. TZ and DQ performed and supervised laboratory tests. RD critically reviewed the paper and contributed to data interpretation and editing of the paper. All authors reviewed and approved the final version.

## Pre-publication history

The pre-publication history for this paper can be accessed here:

http://www.biomedcentral.com/1471-2458/12/533/prepub
